# An Overview of *Helicobacter pylori* VacA Toxin Biology

**DOI:** 10.3390/toxins8060173

**Published:** 2016-06-03

**Authors:** Nora J. Foegeding, Rhonda R. Caston, Mark S. McClain, Melanie D. Ohi, Timothy L. Cover

**Affiliations:** 1 Department of Cell and Developmental Biology, Vanderbilt University School of Medicine, Nashville, TN 37232, USA; nora.j.foegeding@vanderbilt.edu (N.J.F.); melanie.ohi@vanderbilt.edu (M.D.O.); 2 Department of Pathology, Microbiology and Immunology, Vanderbilt University School of Medicine, Nashville, TN 37232, USA; rhonda.r.caston@vanderbilt.edu; 3 Department of Medicine, Vanderbilt University School of Medicine, Nashville, TN 37232, USA; mark.s.mcclain@vanderbilt.edu; 4 Center for Structural Biology, Vanderbilt University, Nashville, TN 37232, USA; 5 Veterans Affairs Tennessee Valley Healthcare System, Nashville, TN 37212, USA

**Keywords:** bacterial toxins, vacuolating toxin, autotransporter, type V secretion, gastric cancer

## Abstract

The VacA toxin secreted by *Helicobacter pylori* enhances the ability of the bacteria to colonize the stomach and contributes to the pathogenesis of gastric adenocarcinoma and peptic ulcer disease. The amino acid sequence and structure of VacA are unrelated to corresponding features of other known bacterial toxins. VacA is classified as a pore-forming toxin, and many of its effects on host cells are attributed to formation of channels in intracellular sites. The most extensively studied VacA activity is its capacity to stimulate vacuole formation, but the toxin has many additional effects on host cells. Multiple cell types are susceptible to VacA, including gastric epithelial cells, parietal cells, T cells, and other types of immune cells. This review focuses on the wide range of VacA actions that are detectable *in vitro*, as well as actions of VacA *in vivo* that are relevant for *H. pylori* colonization of the stomach and development of gastric disease.

## 1. Introduction

*Helicobacter pylori* was first cultured from human gastric tissue in 1983 [1]. Several years later, it was reported that *H. pylori* broth culture supernatants contained a proteinaceous component known as “vacuolating cytotoxin”, which, when added to cultured eukaryotic cells, caused the cells to become vacuolated [2]. Bacterial toxins with similar activity had not been described previously, and initially there was controversy about whether or not a *H. pylori* vacuolating toxin actually existed. Subsequent studies revealed the identity of the vacuolating toxin (termed VacA) [3,4,5,6,7] and showed that it has properties and activities substantially different from those of other bacterial toxins.

## 2. Features of *vacA* and Related Genes in *Helicobacter* Species

**Figure 1:**
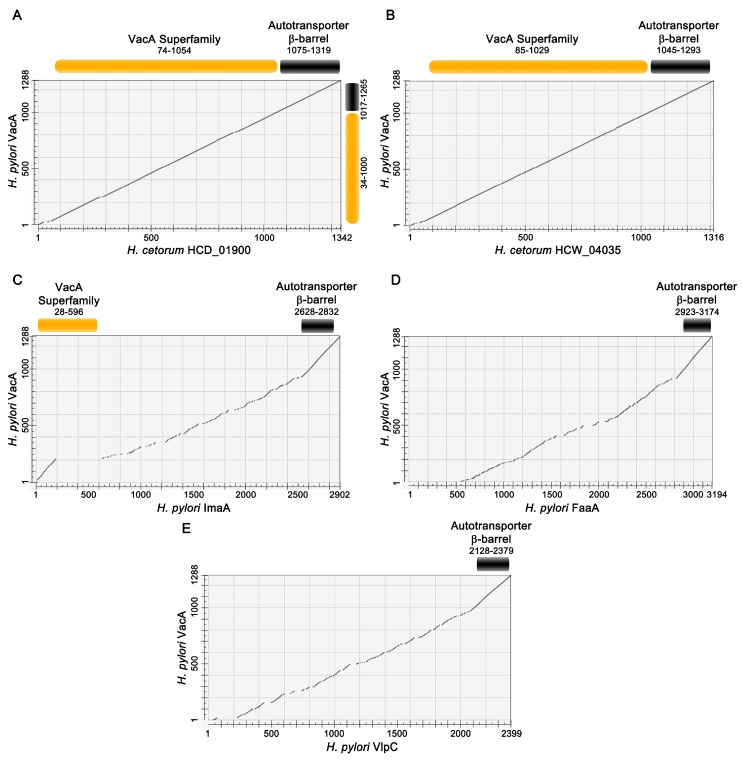
Relatedness of *H. pylori* VacA to *H. cetorum* VacA proteins and *H. pylori* VacA-like proteins. The amino acid sequence of VacA (WP_000405515) from a representative *H. pylori* strain (J99) was aligned to the sequences of related proteins using Needleman–Wunsch global alignment. The amino acid numbers of each protein are shown on the x and y axes, and results of the pairwise alignments are represented as dot matrices. Protein sequences also were searched against the Conserved Domain Database, and domains shared between VacA and the query protein sequence are shown above the dot matrices. Query proteins were *H. cetorum* HCD_01900 (WP_014658932) (**A**); *H. cetorum* HCW_04035 (WP_014660950) (**B**); *H. pylori* ImaA (WP_000808594) (**C**); *H. pylori* FaaA (WP_000222280) (**D**); and *H. pylori* VlpC (WP_000874591) (**E**). The sequences of the latter three *H. pylori* VacA-like proteins are from *H. pylori* strain J99. “VacA superfamily” corresponds to the VacA passenger domain, and “autotransporter β-barrel” corresponds to a domain predicted to be localized to the outer membrane. The autotransporter β-barrel is conserved in all of the proteins analyzed, but there is very little sequence relatedness when comparing the passenger domain of *H. pylori* VacA with corresponding regions of *H. pylori* VacA-like proteins.

All *H. pylori* strains contain a single chromosomal *vacA* gene. The intact *H. pylori* *vacA* gene encodes a protein about 140 kDa in mass. The genus *Helicobacter* includes at least 20 different species, but intact *vacA* genes are present only in *H. pylori* and *H. cetorum*, a species isolated from stomachs or fecal contents of marine mammals [8]. Similar to the association between *H. pylori* and gastritis in humans, *H. cetorum* is associated with gastritis in cetaceans and perhaps pinnipeds (seals) [9,10]. Genome sequence analysis of *H. cetorum* strains from a dolphin and a whale revealed the presence of an intact *vacA* gene next to *cysS* [8], consistent with the linkage of *vacA* and *cysS* in *H. pylori*. The proteins encoded by these *H. cetorum* *vacA* genes exhibit about 60%–70% protein-level identity to the most closely related *H. pylori* *vacA* gene product, and about 66% identity to one another (Figure 1A,B). The dolphin isolate of *H. cetorum* also contains an extra triplet of divergent *vacA* genes [8]. It is not yet known whether *H. cetorum* VacA proteins elicit cytotoxic effects similar to those described for *H. pylori* VacA.

Fragmented *vacA* pseudogenes are found in *H. acinonychis*, a *Helicobacter* species isolated from cheetahs and other large cats [11,12]. Whole genome sequencing of one strain revealed the presence of two nearly identical *vacA* pseudogenes [12]. The gene duplication presumably occurred after the *vacA* gene was disrupted. A protein encoded by a precursor of the *H. acinonychis vacA* pseudogene has been reconstructed *in silico* and exhibits about 64% amino acid identity to its closest match in *H. pylori* [13].

*H. pylori* and several other *Helicobacter* species (including *H. bilis*, *H. heilmannii*, *H. ailurogastricus*, *H. felis*, *H. bizzozeronii*, *H. suis*, *H. acinonychis*, and *H. cetorum*) contain “*vacA*-like” genes. This designation is a bit of a misnomer as the similarity between VacA and these “VacA-like” gene products is limited mainly to the C-terminal ends of the proteins (Figure 1C–E). This C-terminal region is required for secretion of *H. pylori* VacA [5,6,14], but is not part of the soluble VacA toxin. To our knowledge, the three *vacA*-like genes from *H. pylori* are the only *vacA*-like genes that have been studied experimentally. In *H. pylori*, these genes are designated *imaA* (immunomodulatory autotransporter A, HP0289), *faaA* (flagella-associated autotransporter A, HP0609/0610), and *vlpC* (VacA-like protein C, HP0922) [15,16]. Each of the *vacA*-like genes enhances the capacity of *H. pylori* to colonize the stomach in rodent models [15,16,17,18], and transcription of each gene is upregulated in the gastric environment compared to the level of transcription during bacterial growth *in vitro* [15,16,19]. The *H. pylori* VacA-like proteins localize to the bacterial surface, where ImaA and VlpC localize to a bacterial pole and FaaA localizes to the flagella [15,16,20]. Little is known about the functions of these three proteins, but analysis of mutant strains has provided clues. Specifically, analyses of a *faaA* mutant revealed mislocalization of the flagella and decreased bacterial motility [16]; gastric epithelial cells co-cultured with an *imaA* mutant produce higher levels of IL-8 and TNF-α than cells co-cultured with wild-type *H. pylori* [15]; mutations in *vlpC* have been associated with high-level resistance to metronidazole [21].

## 3. VacA Transcription, Regulation, and Secretion

The *H. pylori* *vacA* transcriptional start site is located about 120 nucleotides upstream from the ATG start codon [5,22]. A stem-loop structure in the 5′ untranslated region (UTR) of the *vacA* transcript stabilizes the *vacA* mRNA, particularly during conditions of environmental stress [23]. The transcription of *vacA* is regulated in response to growth phase, with the highest levels of transcription occurring in late log phase [24,25]. There has been relatively little in-depth analysis of *vacA* regulation in response to environmental conditions, but some studies suggest that *vacA* transcription is regulated in response to low pH, iron concentration, salt concentration, and bacterial contact with host cells [23,26,27,28].

**Figure 2:**
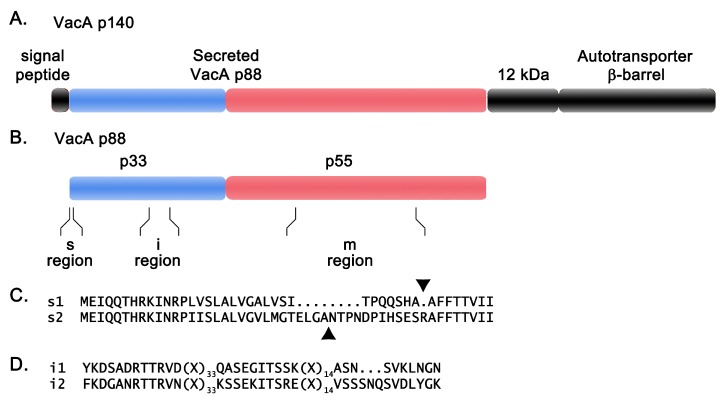
VacA organization and genetic diversity. (**A**) The organization of the 140 kDa VacA protein is shown, including the amino-terminal signal peptide, the secreted 88 kDa VacA toxin, a 12 kDa peptide of unknown function, and a carboxy-terminal domain with a predicted β-barrel structure (autotransporter β-barrel); (**B**) The 88 kDa secreted VacA toxin can undergo proteolytic cleavage into two domains, p33 and p55 (colored **blue** and **red**). Three regions of sequence diversity (s-, i-, and m-regions) are shown; (**C**) Representative signal peptides from type s1 and s2 VacA proteins are shown. Arrowheads mark the sites of signal peptide cleavage. Secreted type s2 VacA proteins contain an amino-terminal extension relative to the secreted type s1 VacA proteins; (**D**) Representative i-region sequences are shown.

VacA is translated into a 140 kDa protein, which undergoes Sec-dependent cleavage of an amino-terminal signal sequence and carboxy-terminal proteolytic cleavage [4,5,6] (Figure 2A). Cleavage yields an active toxin about 88 kDa in mass, as well as a ~12 kDa peptide and a ~33 kDa protein with a predicted β-barrel structure (autotransporter β-barrel) [3,4,5,6,29,30]. The protease responsible for carboxy-terminal proteolytic cleavage has not been identified. The 88 kDa toxin molecules are translocated across the outer membrane and then can be either released into the extracellular space as soluble proteins (along with the 12 kDa peptide) [3,25,29,30] or retained on the bacterial surface [20,31]. The 33 kDa autotransporter β-barrel localizes to the outer membrane, and is required for secretion of the 88 kDa toxin [6,14]. These features suggest that VacA is secreted by an autotransporter or type V mode of secretion [4,5,14].

## 4. Membrane Channel Formation by VacA

Many bacterial toxins enter host cells and cause alterations by exerting an enzymatic activity in intracellular sites, and others act by forming pores in the plasma membrane of host cells. VacA enters host cells, but is not known to have any enzymatic activity. Based on its capacity to form anion-selective channels in planar lipid bilayers [32,33,34], VacA has been classified as a pore-forming toxin. VacA channels can conduct chloride, bicarbonate, and small organic molecules [32,33,34,35,36], and have properties similar to those of ClC channels in host cells [37]. VacA mutant proteins lacking the ability to form a membrane channel in artificial bilayers lack vacuolating toxic activity in cell culture assays [38,39]. Similarly, chemical inhibitors of chloride channels impair VacA channel activity in lipid bilayers and impair vacuolation in cell culture [33,35,36]. Notably, the chemical inhibitors used for these experiments might not act exclusively on VacA channels, but might also act on other cellular targets. Most evidence indicates that the effects of VacA on host cells are directly attributable to channel formation by VacA, rather than activation of endogenous cellular channels.

## 5. VacA Structure

The 88 kDa secreted VacA protein can undergo limited proteolysis in the presence of trypsin or during prolonged storage to yield 33 kDa and 55 kDa fragments (Figure 2B) [6,40]. These are considered to be two domains of VacA (p33 and p55). Mixtures of recombinant p33 and p55 proteins can reconstitute a functionally active form of VacA [41,42]. The p55 domain has a predominantly β-helical structure [43], which is a feature shared by the passenger domains of several proteins secreted by an autotransporter mechanism in other Gram-negative bacterial species [44]. No high resolution structural data are available for the p33 domain. The amino acid sequence of the 88 kDa secreted VacA protein is not closely related to sequences of any other known bacterial toxins.

Both p33 and p55 are required for efficient binding of the toxin to the plasma membrane when VacA is added externally to cells [41]. The p33 domain is required for insertion of VacA into membranes to form anion-selective channels [38,39]. When expressed intracellularly, the minimal VacA region required for cell vacuolation encompasses residues 1–422, which includes the entire p33 domain plus 111 amino acids from the amino-terminal portion of the p55 domain [45,46,47].

The *N*-terminus of the p33 domain contains a sequence of 32 uncharged amino acids, corresponding to the only predicted hydrophobic segment within VacA long enough to span a membrane [38,39,48]. Deletion of this region results in a VacA mutant lacking cell-vacuolating activity and defective in membrane channel formation in planar lipid bilayers [38]. The amino-terminal hydrophobic region of VacA includes three tandem GXXXG transmembrane association motifs (defined by glycine residues at positions 14, 18, 22, and 26) [39,48,49]. Mutagenesis of amino acids within this region, including glycine residues at positions 14 and 18 or a proline residue at position 9, abolishes VacA channel formation and vacuolating activity [39,50,51].

**Figure 3:**
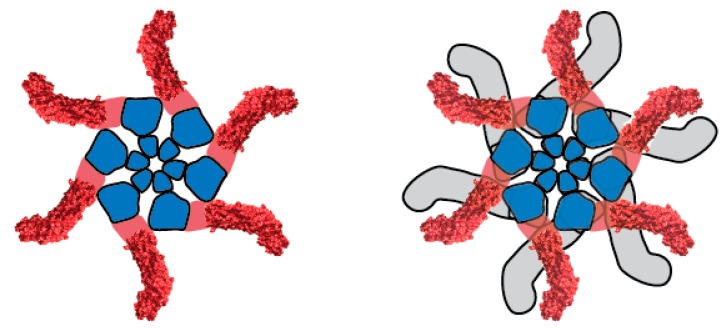
Structural organization of water-soluble VacA oligomers. A hexamer (**left**) and dodecamer (**right**) are shown. Within each component p88 monomer, p33 and p55 domains are shown in blue and red, respectively. A crystal structure has been solved for a portion of p55 [43], corresponding to peripheral elements of the oligomer [54]. Water-soluble hexamers are predicted to be structurally similar to membrane channels formed by VacA.

VacA oligomerizes in solution to form an assortment of flower- or snowflake-shaped structures [52,53,54]. These include double-layered structures (dodecamers and tetradecamers) as well as single layered structures (mainly hexamers and heptamers, but occasionally higher order forms) (Figure 3). Several lines of evidence indicate that oligomerization is required for VacA activity [38,55,56,57,58]. The structure of water-soluble, single layered VacA oligomers is proposed to resemble the structure of VacA membrane channels.

Exposure of VacA oligomers to acidic pH or alkaline pH results in the disassembly of VacA oligomers into monomers [59,60]. When added to cultured cells, preparations of VacA exposed to low pH or high pH have greatly increased cytotoxic activity compared to preparations of oligomeric VacA [61,62]. Therefore, it is thought that VacA first interacts with the plasma membrane of host cells as a monomer, where it oligomerizes and inserts to form a functional membrane channel.

## 6. VacA Diversity among *H. pylori* Strains

Early studies noted that there is considerable variation in vacuolating toxin activity among *H. pylori* strains [2]. One contributing factor is variation among strains in VacA transcription [63]. There is also variation among strains in levels of VacA protein secretion [3]. At present, it is not known whether differences among strains in levels of VacA secretion are primarily due to differences in VacA transcription, differences in transcript stability, or differences in the efficiency with which various forms of VacA are secreted.

All *H. pylori* strains contain a *vacA* gene, and in most strains the *vacA* open reading frame is intact. Frameshift mutations in *vacA* are present in a small proportion of isolates [64], resulting in an absence of VacA protein production. Phylogenetic analysis of *vacA* sequences from a large number of *H. pylori* strains has revealed the existence of several distinct groups of *vacA* alleles, several of which have distinct geographical distributions [13]. For example, *vacA* alleles found in many *H. pylori* strains isolated in East Asia are considerably different from the *vacA* alleles typically found in strains isolated in Europe or Africa [13,65]. Divergence among groups of *vacA* alleles is principally due to positively selected sequence changes in the region of *vacA* encoding the p55 domain, and corresponds to surface-exposed sites in the p55 crystal structure [13].

Three main regions of diversity in VacA sequences have been recognized: the signal sequence region (s-region), the intermediate region (i-region), and middle region (m-region) [66,67] (Figure 2B). Within each of these regions, sequences can be classified into two main types (s1 or s2, i1 or i2, and m1 or m2). The s-region of VacA corresponds to the amino-terminal signal sequence and the amino-terminus of the secreted toxin (Figure 2C) [66,68,69,70]. The i-region is found within the p33 domain of VacA (Figure 2D) [67]. The m-region corresponds to part of the p55 domain [66]. Homologous recombination occurs commonly in *H. pylori*, and consequently, *vacA* alleles can contain multiple possible combinations of s-, i- and m-region types (s1-i1-m1; s2-i2-m2; s1-i1-m2, *etc.*) [66].

In contrast to type s1 forms of VacA, type s2 forms of VacA do not cause vacuolation of mammalian cells [66,68,69,70]. This dichotomy in activity is attributable to different sites of signal sequence cleavage in s1 and s2 VacA proteins. Specifically, a hydrophilic 12 amino acid N-terminal extension is present in type s2 proteins, but absent from type s1 proteins [66,68,69,70]. Several studies have reported that type m1 and m2 forms of VacA exhibit distinct cell-type specificities. For example, the activity of type m1 VacA toward HeLa cells is greater than the activity of type m2 VacA toward these cells, whereas both m1 and m2 forms are highly active toward RK-13 cells [71,72]. The difference in activity of m1 and m2 VacA proteins has been attributed to cell-type dependent variations in VacA binding to cells [71], and may also be attributable to differences in channel-forming properties [73]. Cell type specificity has been mapped to a 148-residue segment within the m-region of VacA [72]. One study reported that the i-region (within the p33 domain) is a determinant of vacuolating toxin activity in strains that produce type s1-m2 forms of VacA [67]. Type i1 forms of VacA are more active than type i2 forms of VacA in experiments conducted with Jurkat T cells [74].

Epidemiological studies have demonstrated a correlation between the type of *vacA* allele present in *H. pylori* strains and the risk of gastroduodenal disease. Specifically, the risk of gastric cancer or peptic ulcer disease is higher in persons infected with strains containing type s1, i1 or m1 forms of *vacA*, compared to persons infected with strains containing type s2, i2 or m2 forms of *vacA* [66,67,75,76]. The increased risk of disease observed with strains containing s1, i1, or m1 forms of *vacA* is probably attributable not only to the effects of VacA, but also to the effects of additional virulence determinants. Specifically, strains containing a type s1 *vacA* allele typically harbor the *cag* pathogenicity island (which encodes several important virulence determinants, including CagA and a type IV secretion system) and the outer membrane protein adhesin BabA, whereas strains containing a type s2 *vacA* allele typically lack the *cag* pathogenicity island and often lack *babA* [76].

## 7. VacA Activities in Cell Culture Systems

### 7.1. Effects on Epithelial Cells

#### 7.1.1. Endosomal Alterations

The most extensively studied activity of VacA is its ability to cause vacuolation of cultured cells. Most studies of this phenomenon and other VacA activities have been conducted with the highly active s1-i1-m1 form of VacA. Vacuolation can be observed within a few hours after addition of VacA to cells and is enhanced by the presence of weak bases [77]. The membranes of VacA-induced vacuoles contain markers typically found in membranes of late endosomes (LEs) [78,79,80,81], which suggests that the vacuoles arise from late endosomal compartments [82]. The current model for VacA-induced vacuolation [83,84] proposes that the secreted monomeric form of VacA binds to the plasma membrane. Upon binding, VacA monomers form oligomers, which are trafficked to LEs, where they form anion-selective channels in the LE membrane [32,33,34,35,36]. Transit of chloride ions through VacA channels in the LE membrane leads to an increase in intraluminal chloride concentration, which in turn triggers the enhancement of V-ATPase proton pumping activity and a decrease in intraluminal pH. Membrane-permeant weak bases diffuse into LEs, where they are protonated in the acidic environment and trapped. As a result, LEs undergo osmotic swelling, resulting in cell vacuolation [85,86,87]. In addition to causing cell vacuolation, VacA causes a variety of functional alterations related to disruption of proper endocytic compartment trafficking. These include inhibition of intracellular degradation of epidermal growth factor [88], inhibition of procathepsin D maturation [88], perturbation of transferrin recycling [89], and in immune cells, inhibition of antigen presentation [90].

#### 7.1.2. Autophagy

When added to cultured gastric epithelial cells, VacA induces autophagy [91], the regulated degradation and recycling of cellular components in the cytoplasm. VacA is necessary and sufficient for *H. pylori*-induced autophagy [91]. Similar to VacA-induced vacuole formation, VacA-induced autophagy is dependent on the capacity of VacA to form membrane channels [91], but the autophagosomes formed in response to VacA are distinct from the more abundant and larger intracellular vacuoles that form in response to VacA [91]. Although the mechanisms by which VacA induces autophagy are not fully understood, VacA-induced autophagy has been shown to depend on binding of VacA to low-density lipoprotein receptor-related protein 1 (LRP1) [92]. Inhibition of autophagy leads to increased stability of intracellular VacA and increased cell vacuolation [91]. One hypothesis is that the induction of autophagy upon exposure to VacA is a response initiated by the host cell to degrade VacA and prevent additional toxin-induced cell damage [91]. Although acute exposure of host cells to VacA induces autophagy, prolonged exposure of host cells to VacA has been shown to disrupt autophagy [93,94].

#### 7.1.3. Mitochondrial Alterations

Treatment of cells with VacA results in an assortment of mitochondrial alterations, including reduction of mitochondrial transmembrane potential [95,96,97], release of cytochrome *c* [97,98,99], activation of Bax and Bak [97,100], and mitochondrial fragmentation [100]. After entry into host cells, VacA can localize to mitochondria [96,98,100,101], leading to the hypothesis that the toxin acts directly on mitochondria. In support of this model, VacA can cause a reduction in the transmembrane potential of isolated mitochondria [97], and VacA is imported into the inner mitochondrial membrane (IMM) [98,102,103]. The ability of VacA to induce mitochondrial dysfunction is dependent on VacA channel activity [96,99,100]. Thus, one model proposes that VacA is imported into mitochondria and induces mitochondrial transmembrane potential reduction, perhaps by pore formation. This depolarization stimulates an initial release of cytochrome *c*, the activation of Bax/Bak, and the subsequent Bax/Bak-dependent release of cytochrome *c*. Another hypothesis is that VacA-induced mitochondrial dysfunction is due to indirect actions of VacA. For example, VacA may act indirectly by activating pro-apoptotic factors to trigger mitochondrial-dependent apoptosis [97].

#### 7.1.4. Epithelial Barrier Alterations

When added to cultured epithelial cells, VacA causes increased plasma membrane permeability, resulting in efflux of various anions and other small molecules, including chloride, urea, and bicarbonate, into the extracellular space [104,105]. VacA-induced permeabilization of cells is attributed to the formation of VacA channels in the plasma membrane [33,34,36,105]. In addition to causing increased permeability of the plasma membrane, VacA causes increased paracellular permeability of polarized monolayers [106,107,108]. The mechanism by which VacA causes increased paracellular permeability is not well understood.

#### 7.1.5. Altered Cell Signaling

Several cellular alterations can be detected very rapidly after exposure of cells to VacA, and are likely due to the binding of VacA to the surface of host cells. In both gastric epithelial cells [109,110] and T cells [111], VacA activates p38, a mitogen-activated protein (MAP) kinase. VacA-induced activation of the p38 signaling pathway leads to induction of cyclooxygenase 2 (COX-2) expression, which results in enhanced prostaglandin E2 production [110]. VacA-induced activation of the p38 signaling pathway also can lead to the activation of activating transcription factor 2 (ATF-2) [109]. VacA can also activate another MAP kinase, ERK1/2 [109]. In addition to activating MAP kinases, VacA can activate a signaling pathway that activates G protein-coupled receptor kinase interactor (Git1) [112], a signaling pathway that leads to the upregulation of vascular endothelial growth factor (VEGF) [113], and the β-catenin signaling pathway [114]. The VacA cell surface receptors required for activating most of these pathways have not been characterized, but RPTP-β is reported to be the VacA receptor required for activation of Git1 [112] and epidermal growth factor receptor is reported to be required for upregulation of VEGF [113].

#### 7.1.6. Cell Death

VacA-induced cell vacuolation is not a cytolethal alteration [2], but exposure of epithelial cells to VacA can potentially result in cell death [101,115,116]. AZ-521 cells (which are of duodenal origin) are particularly susceptible to VacA-induced cell death [117,118]. VacA-induced cell death is preceded by an assortment of mitochondrial alterations [95,96,97,98,99,100,119], which suggests that these alterations are mechanistically important in the process by which VacA causes cell death. VacA reduces the expression of pro-survival factors [120] and causes endoplasmic reticulum (ER) stress [121], which could also contribute to VacA-induced cell death. VacA can cause cell death by both apoptosis and necrosis [117].

### 7.2. Effects on Immune Cells and Parietal Cells

#### 7.2.1. Effects on Immune Cells

VacA can alter the function of many types of immune cells [84,122], including lymphocytes, macrophages, eosinophils [123,124], mast cells [125,126], and dendritic cells [127,128]. VacA inhibits activation and proliferation of T cells and B cells [111,129,130,131], and can interfere with antigen presentation in B cells [90]. In macrophages, VacA contributes to the formation of large vesicles termed megasomes, and impairs the maturation and function of vesicular compartments [132,133]. VacA alters various signal transduction pathways in macrophages [134,135], and can cause macrophage apoptosis [136]. These VacA-induced effects may impair the ability of macrophages to engulf *H. pylori.* In addition to immunosuppressive effects, VacA stimulates the expression of the proinflammatory enzyme COX-2 in macrophages and neutrophils [111].

#### 7.2.2. Effects on Parietal Cells

Two studies reported that VacA inhibits gastric acid secretion from parietal cells [137,138]. In one study, exposure of parietal cells to VacA resulted in permeabilization of the plasma membrane and calcium influx that ultimately caused the disruption of actin arrangement in apical microvilli and an inhibition of acid secretion [138]. At present, it is not known whether this effect of VacA on parietal cells contributes to a reduction in gastric acid secretion that is sometimes observed in the course of *H. pylori* infection.

## 8. Binding, Internalization, and Intracellular Trafficking of VacA

### 8.1. Cell Surface Binding and Receptors

Various studies have reached differing conclusions about whether VacA binding to cells is saturable [139,140,141] or nonsaturable [62,142,143]. Therefore, it is unclear whether VacA binds to a single abundant, low-affinity receptor or to multiple cell surface components. Multiple putative VacA receptors on the surface of epithelial cells have been reported, including both protein and lipid receptors. These include receptor protein tyrosine phosphatases (RPTP) α and β [60,112,144,145], low-density lipoprotein receptor-related protein-1 (LRP1) [92], epidermal growth factor receptor (EGFR) [146], heparan sulphate [147], sphingomyelin [148,149], glycosphingolipids [150], and phospholipids [151]. Among these putative receptors, sphingomyelin is the only plasma membrane component whose presence or absence is a functionally important determinant of sensitivity of epithelial cells to VacA, and also an important determinant of the extent to which VacA binds to the cell surface [148]. Binding of VacA to sphingomyelin probably accounts for localization of the toxin to lipid rafts [143,152,153]. Although several putative receptors for VacA have been identified on epithelial cells, β2 integrin subunit (CD18) is the only VacA receptor that has been identified on T cells [154].

VacA binding to RPTP-β triggers alterations in cell signaling that ultimately lead to gastric tissue damage [112]. Correspondingly, oral administration of VacA to wild-type mice results in gastric damage, whereas RPTP-β knockout mice are resistant to VacA-induced gastric damage [112]. RPTP-β is not the sole receptor for VacA, as VacA is still internalized into epithelial cells in RPTP-β knockout mice [112]. VacA binding to LRP1 is important for VacA-induced autophagy and apoptosis [92].

### 8.2. Pore Formation at the Cell Surface

After binding to the cell surface, VacA increases plasma membrane permeability and causes membrane depolarization [36,39]. These alterations are attributed to insertion of VacA into the plasma membrane and formation of anion-selective membrane channels [32,33,34,35,36]. It is proposed that the toxin can also form channels in intracellular sites (endosomes and mitochondria). Relatively little is known about the relationships between VacA channel formation and intracellular trafficking of the toxin.

### 8.3. VacA Internalization and Intracellular Trafficking

Upon binding to the cell surface, VacA is internalized by a clathrin-independent, Cdc42 dependent, and Rac1 dependent route that requires actin polymerization [155,156,157,158]. Within 10 min after internalization, VacA is found in glycosylphosphatidyl inositol anchored protein (GPI-AP)-enriched early endosomal compartments (GEECs), within 30 min in early endosomes (EEs), and within 2 h in LEs [156,157].

Several studies have provided evidence that the intracellular localization of VacA is not limited to endosomal compartments. As one example, VacA has been detected in association with mitochondria in host cells [96,119]. The mechanisms by which VacA traffics to mitochondria are not well understood. One model proposes that a subset of VacA-containing endosomes co-localize with mitochondria, and that VacA is transferred directly from endosomes to mitochondria [119]. In support of this model, VacA causes cellular changes that result in the co-fractionation of endosomes with mitochondria [119]. Another model proposes that VacA is released into the cytosol and is imported into mitochondria via mitochondrial import proteins [102,103]. Although VacA gains access to the cytosol if expressed in host cells or microinjected into cells [45,98], it is not known whether VacA added externally to cells can ultimately gain access to the cytosol (either by directly crossing the plasma membrane or by release from endosomes). It has been suggested that VacA may travel retrograde through the Golgi and ER [81], but this has not been investigated in detail. Further studies are needed to better understand VacA trafficking within host cells.

## 9. Activity of VacA in Animal Models

Animal models have been utilized to investigate a potential role of VacA in promoting *H. pylori* colonization of the mammalian stomach. VacA mutant strains of *H. pylori* can colonize mice, gerbils, and gnotobiotic piglets [159,160,161,162], which indicates that VacA is not essential for gastric colonization. However, when coinfections are performed with isogenic wild-type and VacA mutant strains, VacA mutant strains are at a competitive disadvantage [162,163]. In addition, Δ*vacA* mutant strains colonize mice less efficiently or at reduced levels compared to wild-type strains [128,162,163].

Experiments in animal models have also revealed a role for VacA in promoting gastric pathology. Oral or intragastric administration of purified VacA to mice results in damage to the gastric mucosa and recruitment of inflammatory cells [6,112,125,164]. Based on the capacity of VacA to induce ulceration in mice when administered intragastrically, it has been suggested that VacA contributes to the pathogenesis of gastric ulceration in humans. The intragastric concentration of VacA in animals receiving intragastrically administered VacA substantially exceeds the 20–800 pg per mL concentration of VacA estimated to be present in gastric juice, based on a bead-based ELISA performed on samples from *H. pylori*-positive patients [165], but local concentrations of VacA at sites of *H. pylori* interaction with gastric epithelial cells might be considerably higher than concentrations in gastric juice. In studies of gerbils experimentally infected with *H. pylori*, *vacA* mutant strains are less likely than isogenic wild-type strains to produce gastric ulcers [161]. In addition, colonization studies in mice indicate that *H. pylori* strains producing forms of VacA that are most active *in vitro* (s1-i1) induce more severe and extensive metaplasia and inflammation in the stomach than strains producing forms of VacA that are less active *in vitro* (s1-i2 or s2-i2) [163]. Another study showed that, in comparison to a wild-type strain, Δ*vacA* mutants induce stronger T-helper 1 (Th1) and T-helper 17 (Th17) responses and trigger more severe gastric pathology in mice [128]. This latter observation may be attributable to immunomodulatory activities of VacA.

*H. pylori*-infected humans often develop serum and gastric mucosal antibody responses to VacA (as well as many other *H. pylori* antigens) [166,167], but these humoral immune responses do not result in clearance of *H. pylori* infection. In contrast, immunization of animals with VacA provides protective immunity against subsequent challenge with *H. pylori* [168,169,170,171,172,173]. VacA has also been used as a component of vaccines used for therapeutic immunization (*i.e.*, immunization designed to promote clearance of *H. pylori* infection) [169,171,173].

The presence of *H. pylori* in humans is inversely correlated with the incidence of allergy and asthma [174,175]. Studies in mice have shown that *H. pylori* infection protects against development of allergic asthma, and that the protective effect of *H. pylori* is attributed to tolerogenic reprogramming of dendritic cells [176]. VacA (and also gamma-glutamyl transpeptidase, GGT) contribute to the ability of *H. pylori* to induce tolerizing effects on murine dendritic cells *in vitro* and *in vivo* [128,177] and VacA is required for protection against allergic asthma in a mouse model [128,177].

## 10. Comparisons of VacA Activities *in Vitro* and *in Vivo*

VacA causes a wide spectrum of alterations in multiple cell types *in vitro*. One might presume that some VacA activities observed *in vitro* are more relevant *in vivo* than others. Therefore, there is considerable interest in defining VacA activities that are most relevant *in vivo* for promoting *H. pylori* colonization of the stomach or the development of diseases such as peptic ulceration or gastric cancer.

Cell vacuolation is one of the most extensively studied VacA-induced phenomena *in vitro*. Human gastric epithelial cells are susceptible to VacA [178,179] and vacuolation is occasionally observed in epithelial cells from gastric biopsies [180], albeit less prominently than in cultured cells treated with VacA. One study reported that VacA can promote intracellular survival of *H. pylori* in gastric epithelial cells [181]. Conversely, *H. pylori* is predominantly an extracellular organism and does not replicate within intracellular vacuoles [182]. Therefore, it is difficult to envision a mechanism by which vacuole formation *per se* would be relevant for *H. pylori* colonization of the stomach or *H. pylori*-associated gastric diseases. On the other hand, alterations in host cells resulting from VacA-induced perturbation of endocytic trafficking could be advantageous for the bacteria (for example, by inhibiting antigen presentation) [90].

One plausible action for VacA *in vivo* is to enhance the availability of nutrients or essential growth factors (such as metals), thereby promoting growth of *H. pylori*. Nutrients could be released through VacA channels in the plasma membrane or by VacA-induced changes in paracellular permeability. One study provided evidence that VacA perturbs transferrin recycling in host cells, leading to enhanced availability of iron and enhanced growth of *H. pylori* on the surface of epithelial cells [89]. VacA-induced cell death could also result in release of nutrients.

VacA interferes with the functions of several types of immune cells *in vitro*, and this could also be an important action of the toxin *in vivo*. Specifically, by interfering with the normal functions of T cells, B cells, neutrophils, macrophages, and eosinophils, VacA may attenuate the host immune response and thereby facilitate persistent *H. pylori* colonization of the stomach.

*H. pylori* strains producing s1 forms of VacA, which are most active *in vivo*, typically contain the *cag* pathogenicity island, whereas strains producing s2 forms of VacA often lack the *cag* PAI [66]. The *cag* PAI encodes an effector protein, CagA, which causes numerous alterations in host cells, as well as a type IV secretion system required for entry of CagA into host cells. The co-selection of type s1 *vacA* and the *cag* PAI, as well as similarities in the phylogenetic structure of *vacA* and *cagA* genes [13], suggests the existence of a functional interaction between VacA and products of the *cag* PAI. In support of this hypothesis, several studies have shown that VacA partially inhibits the actions of CagA *in vitro*, and CagA partially inhibits the trafficking or actions of VacA [101,183,184,185,186,187]. Thus, another important action of VacA *in vivo* may be to provide an optimal counterbalance for the actions of CagA.

## 11. Summary

In summary, VacA is a secreted bacterial toxin that differs substantially from other known toxins in amino acid sequence, structure, intracellular trafficking, and actions. Many different cell types are susceptible to VacA *in vitro*, and the toxin can cause a wide spectrum of cellular alterations. *In vivo*, VacA enhances the capacity of *H. pylori* to colonize the stomach and contributes to the pathogenesis of *H. pylori*-induced diseases. In future studies, it will be important to define more clearly the mechanisms by which VacA causes alterations in host cells and to define the actions of this toxin that are most relevant *in vivo*.
